# Granuloma cells in chronic inflammation express CD205 (DEC205) antigen and harbor proliferating T lymphocytes: Similarity to antigen-presenting cells

**DOI:** 10.1111/pin.12036

**Published:** 2013-02-22

**Authors:** Haruo Ohtani

**Affiliations:** Department of Pathology, Mito Medical Center, National Hospital OrganizationIbaraki, Japan

**Keywords:** antigen presenting cells, CD205 (DEC205), granuloma, granulomatous inflammation, T cells

## Abstract

Granulomas are classified as immune or foreign body granulomas. Of these, the immune granulomas, a hallmark of granulomatous inflammation, are closely related to cell-mediated immune responses. The aim of the present study is to characterize immune granuloma cells in 33 patients with granulomatous inflammation focusing on the expression of CD205 (DEC205), a cell surface marker of antigen presenting cells, and their spatial relationship to T cells. CD205 was frequently expressed by immune granuloma cells, in contrast to foreign body granuloma cells that lacked CD205 expression. T cells were not only distributed in a lymphocyte collar around the granuloma, but also present among the granuloma cells (termed ‘intra-granuloma T cells’). Intra-granuloma T cells stained positive for Ki-67 (median positivity = 9.4%) by double immunostaining for CD3 and Ki-67. This indicated the presence of proliferative stimuli within the granuloma that could activate the intra-granuloma T cells. The labeling index of Ki-67 in intra-granuloma T cells was significantly higher than that of T cells in the lymphocyte collar (*P* < 0.0001) or T cells in the T cell zone (paracortex) of chronic tonsillitis or reactive lymphadenitis (*P* = 0.002). These data indicate a close similarity between immune granulomas and antigen presenting cells.

Granulomas are composed of a collection of epithelioid granuloma cells and giant cells, which are frequently surrounded by a lymphocyte collar. Granulomas are classified as immune or foreign body granulomas.^1^ Granulomatous inflammation, characterized by the formation of immune granulomas associated with lymphocytic infiltrates, is observed in tuberculosis, leprosy, syphilis, cat-scratch disease, sarcoidosis, Crohn's disease and some mycotic infections. In these types of inflammation, granulomas are traditionally thought to confine offending agents that are difficult to eradicate.^1^ The cellular components of immune granulomas include macrophages, T cells (including T helper type 1 cells [Th1], T helper 17 cells [Th17], regulatory T cells [Treg], and CD8^+^ T cells), B cells and dendritic cells (DCs).^2^–^5^ In these immune granulomas, T cell-mediated immune responses have been shown to occur. Interestingly, granuloma cells may be derived from macrophages^1^ and classically activated macrophages^3^ or M1 macrophages^6^ can promote T cell responses.

Given the presence of active T-cell mediated immune responses in granulomatous inflammation, it may be reasonable to consider granuloma cells as linking the innate and adaptive immune responses. We previously demonstrated that granuloma cells in Crohn's disease expressed CD86 (B7-2), CD80 (B7-1) and HLA-DR, and observed direct cell-to-cell contact between granuloma cells (including giant cells) and lymphocytes by immunoelectron microscopy.^7^ Furthermore, granuloma cells in Crohn's disease expressed CC-chemokine ligand 5 (CCL5 also known as RANTES) and T cells distributed around the granuloma expressed CC-chemokine receptor 5 (CCR5), a cognate receptor for CCL5.^8^ These data are in accordance with the view that immune granuloma cells may function as antigen presenting cells (APCs). To further investigate this concept, the present study analyzed the expression of CD205 (DEC205) antigen in various kinds of granulomatous inflammation. CD205 is an endocytic receptor homologous to the macrophage mannose receptor, which is important for antigen presentation to T cells. It is expressed by DCs and thymic epithelial cells.^9^,^10^ In human tissue, immunohistochemistry CD205 has been used to identify DCs in the tonsils^11^ and spleen.^12^ CD205 expression is up-regulated upon maturation of human monocyte-derived dendritic cells.^13^ Alveolar macrophages in mouse and human lung tuberculosis tissues also express CD205.^14^,^15^ CD205 is also expressed by activated human plasmacytoid DCs, another component of major subsets of DCs (i.e. the other component of mouse and human DCs is myeloid DCs [also known as conventional DCs and classical DCs]).^16^

The present study demonstrates that immune granuloma cells from various granulomatous inflammations frequently express CD205 at greater levels than previously reported,^14^,^15^ and observed that the granuloma cells consistently harbor T cells (termed ‘intra-granuloma T cells’), which express high levels of Ki-67, a proliferation-associated antigen. The author discusses the significance of this similarity between granuloma cells and APCs.

## Materials and Methods

### Patient samples and materials

Thirty three cases with granulomatous inflammation were analyzed (Table 1), which included tuberculosis (*n* = 10), urothelial carcinoma treated by bacillus Calmette-Guérin (BCG) (*n* = 6), granulomatous reaction in lymphocyte-rich cancer stroma (*n* = 4), Crohn's disease (*n* = 2), granulomatous prostatitis (cause unidentified) (*n* = 2) and one case each of primary biliary cirrhosis, sarcoidosis (lung), subacute thyroiditis, granulomatous lobular mastitis, extrinsic allergic alveolitis, sarcoid-like reaction in patient with bile duct adenocarcinoma, granulomatous dermatitis (cause unidentified), granulomatous pleuritis (cause unidentified) and granulomatous inflammation of the lung associated with lung cancer (cause unidentified). In patients with non-malignant diseases, the sampling was performed to confirm the diagnosis, and no treatments had been performed on the patients. In patients with malignancy, sampling was performed for the purpose of treatment, and no treatments had been performed except for patients with BCG-treated urinary bladder carcinoma. Control samples for granulomatous inflammation included 14 cases of foreign body granulomas without pre-operative treatments (11 cases of epidermal cyst, one case of calcifying epithelioma, one case of skin foreign body reaction and one case of foreign body reaction in the submandibular gland). In addition, 11 cases of chronic tonsillitis and five cases of reactive hyperplasia of lymph nodes (reactive lymphadenitis) were used as controls of reactive changes of the T cell zone (paracortex) of the lymphoid tissue (16 cases in total). Nearly all patients with chronic tonsillitis had been administered with antibiotics. However, this would not affect the results significantly since all the tonsillitis tissues showed marked hyperplasia of lymphoid tissues. The average ages (± 1 standard deviation), age ranges and male to female ratios in granulomatous inflammation, foreign body granulomas and lymphoid tissues were 65.2 ± 13.1 (22–86) with M:F = 22:11, 52.4 ± 17.0 (23–78) with M:F = 11:3, and 35.2 ± 20.7 (5–79) with M:F = 6:10, respectively. All the samples were selected from the archives of histopathological diagnosis in our Hospital, diagnosed from January 2009 to April 2012. All samples were obtained either by surgery or biopsy, fixed in formalin, and routinely processed for diagnosis. The present study was approved by the Ethics Committee of Mito Medical Center.

**Table 1:** Patient characteristics

Diseases	*n*	Details of patients
Tuberculosis	10	Lung, 3, lymph nodes 3, skin 1, bursa 1, pleura 1, prostate 1
BCG vaccine-induced granulomatous inflammation in urothelial carcinoma of the bladder	6	
Granulomatous reaction in TIL-rich cancer stroma	4	Lung adenocarcinoma 1, gastric adenocarcinoma with lymphoid stroma (lymphoepithelioma-like carcinoma) 1, hepatocellular carcinoma 1, peripheral T-cell lymphoma, NOS 1
Crohn's disease	2	
Primary biliary cirrhosis	1	
Sarcoidosis (lung)	1	
Subacute thyroiditis	1	
Granulomatous lobular mastitis	1	
extrinsic allergic alveolitis	1	
Sarcoid-like reaction of lymph nodes (bile duct adenocarcinoma)	1	
Granulomatous inflammation of prostate (cause unidentified)	2	
Granulomatous pleuritis (cause unidentified)	1	
Granulomatous inflammation of the skin (cause unidentified	1	
Granulomatous inflammation of the lung (cause unidentified)	1	
Total	33	

BCG, bacillus Calmette-Guerin; *n*, number of patients; TIL, tumor-infiltrating lymphocyte.

### Primary antibodies

For immunohistochemistry, the following primary antibodies were used: mouse monoclonal antibody to human CD205 (clone 11A10, IgG1, Leica Microsystems, Benton Lane, UK), CD20 (clone L26, DAKO, Glostrup, Denmark), HLA-DR (clone TAL.1B5, DAKO), CD68 (clone PG-M1, DAKO) and rabbit polyclonal antibody to human CD3 (DAKO).

### Single-labeling immunohistochemistry

After heat antigen retrieval in 10 mM Tris/1 mM ethylene diaminetetra-acetic acid (EDTA) buffer, pH 9.0, for 60 min at 95°C, non-specific binding was blocked using Protein Block (DAKO). The primary antibodies listed above were incubated for 30 min at room temperature. After quenching endogenous peroxidase activity by immersing specimens in 3% H_2_O_2_ solution for 5 min, horseradish peroxidase-labeled Envision plus (DAKO) was applied as the secondary antibody. DAB was used for chromogen.

### Double immunofluorescent staining for CD3 and CD205

Double immunofluorescent staining was performed using formalin-fixed, paraffin embedded sections as previously described.^17^ In brief, after the same pretreatment of the specimens described above, a mixture of mouse monoclonal anti-CD205 (1:400) and rabbit polyclonal anti-CD3 (1:100, 6 μg/mL) was applied overnight at 4°C. A mixture of donkey anti-rabbit immunoglobulin antibody labeled with Alexa Fluor 488 and donkey anti-mouse immunoglobulin antibody labeled with Alexa Fluor 555 was then incubated for 30 min. Red (anti-CD205), green (anti-CD3) and blue (4',6-diamidino-2-phenylindole [DAPI] nuclear stain) fluorescent images were observed, captured and merged with a confocal laser scanning microscope (SP-6, Leica, Wetzler, Germany).

### Double staining for CD3 and Ki-67

Enzyme-linked double immunohistochemistry was performed as previously described.^18^ In brief, the pretreatment of specimens was performed as described above. A mixture of rabbit polyclonal anti-human CD3 antibody (1:50) and mouse monoclonal anti-human Ki-67 antibody (clone MIB1, 1:1000, DAKO) was incubated overnight at 4°C. After quenching endogenous peroxidase activity (as described above), the sections were incubated with anti-rabbit secondary antibody conjugated with alkaline phosphatase (Simple stain, Nichirei, Tokyo, Japan), and then with anti-mouse Envision plus (horseradish peroxidase, DAKO). For color development, Vulcan Fast Red Kit (Biocare, City, Country) and diaminobenzidine Kit (DAKO) were used separately. After hematoxylin counter staining the sections were air-dried and mounted.

### Double staining for CD205 and CD68

A two-step method was used for double staining of CD205 and CD68. First, staining for CD205 was performed as above but with an anti-mouse immunoglobulin secondary antibody conjugated with alkaline phosphatase (simple stain). The specimens were re-retrieved at 95°C for 30 min. Then immunohistochemistry for CD68 was performed using anti-mouse immunoglobulin secondary antibody conjugated with horseradish peroxidase (Envision plus).

### Cell counting of CD3^+^ Ki-67^+^ cells

Cells were counted as previously described.^19^ In brief, specimens for double immunohistochemistry of CD3 and Ki-67 were carefully observed to identify granulomas. The areas of granulomas were observed using a 40 × objective lens, with a microscopic grid of 10 × 10 mm set in an ocular lens with a BX51 microscope (Olympus, Tokyo, Japan). The area for cell counting was 0.0625 mm^2^. The numbers of CD3^+^ Ki67^+^ cells and total CD3^+^ cells in one grid were manually counted. At least three areas were counted for each case. For statistical analysis, SPSS ver. 21 (SPSS, Chicago, IL, USA) was used.

## Results

### CD205 is expressed by immune granuloma cells irrespective of disease type

**Figure 1:**
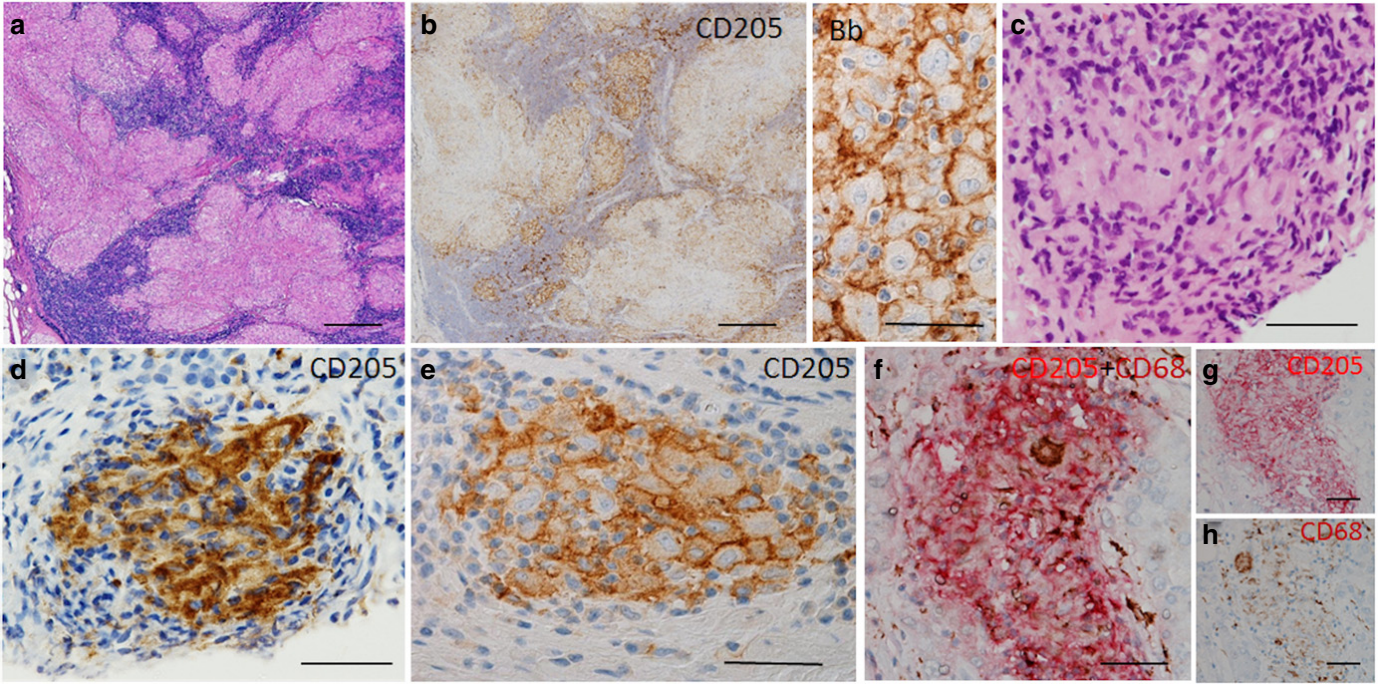
Immunohistochemical expression of CD205 in granuloma cells. (**a**) HE and (**b**) immunostaining for CD205 in immune granulomas in lymph node tuberculosis. Immunoreactivity is expressed by brown color in all single-labeling immunohistochemistry in this paper. (**b**) CD205 is more strongly expressed in small granulomas or in the periphery of large granulomas. High magnification of CD205^+^ granuloma cells (Bb). (**c**) HE (**d**) and immunostaining for CD205 in immune granuloma in primary biliary cirrhosis. (**d**) Note clear, membranous staining of CD205 in granuloma cells. (**e**) Immunostaining for CD205 in a small-sized granuloma in the submucosa of colon of Crohn's disease, showing clear membranous reactivity of CD205. (**f**) Double staining for CD205 (red) and CD68 (brown) in granuloma in hepatocellular carcinoma tissue, revealing that CD68^+^ granuloma cells express CD205. (**g**) Negative control for CD205 only and (**h**) CD68 only. Scale bars: 250 μm in (**a,b**) and 50 μm in (**c–h**).

**Figure 2:**
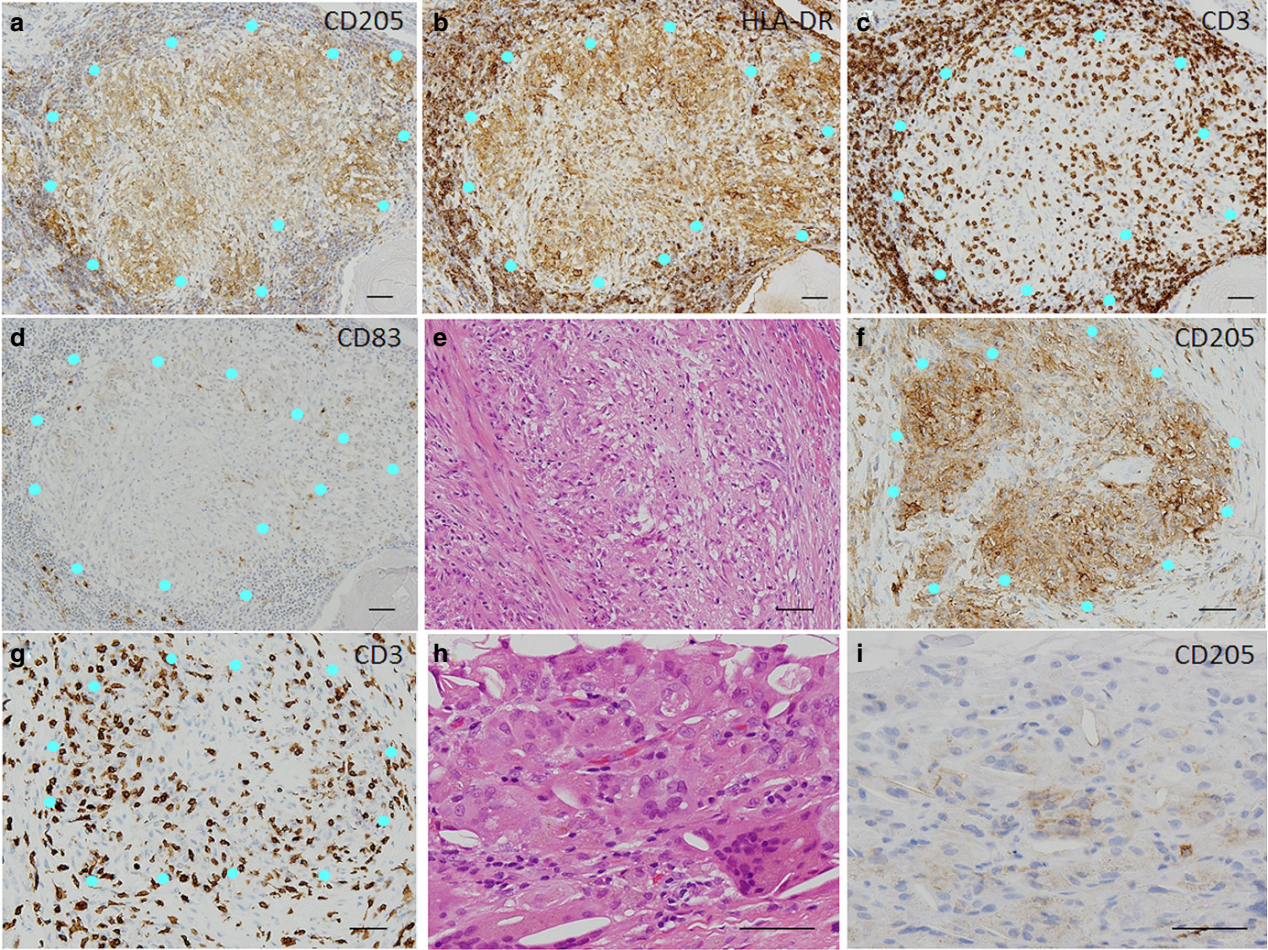
(**a–d**) Immunohistochemical comparison among immune granuloma with lymphocyte collar, (**e–g**) immune granuloma without lymphocyte collar, and (**h,i**) foreign body granuloma. The original disease is (**a–g**) urothelial carcinoma treated with BCG and (**h,i**) epidermal cyst. (**a,f,i**) Immunostaining for CD205, (**b**) HLA-DR, (**c,g**) CD3, (**d**) CD83, and (**e,h**) HE. Note that CD205 is expressed in granuloma (**a**) with- and (**f**) without lymphocyte collar, but not in (**i**) foreign body granuloma cells. (**d**) CD83^+^ mature (conventional) dendritic cells are distributed in the lymphocyte collar, but are sparse among granuloma cells. Intra-granuloma T cells are distributed in granulomas (**c**) with and (**g**) without lymphocyte collar. Blue dots in **a**–**d** and **f**,**g** indicate the area of granuloma cells. Scale bars: 50 μm (**a–i**).

Immunohistochemistry revealed that the expression of CD205 was present as a membranous staining pattern on granuloma cells in all the cases examined (Figs 1A–F, 2A,F,S1B,S2B). In 26 of 33 cases, more than half of granuloma cells expressed CD205 including Langerhans giant cells. No specific results were observed among each disease case. The staining intensity, irrespective of the disease, was usually clearer and stronger in small-sized granulomas compared with large-sized, conglomerated granulomas (Fig. 1A,B). In seven of 33 cases, the expression of CD205 was observed at focal points: 20–50% of granulomas were positive in five cases (two cases of Crohn's disease, one case each of tuberculous lymphadenitis, BCG-treated urothelial carcinoma and lung tuberculosis), and 10–20% of granulomas were positive in one case of BCG-treated urothelial carcinoma and sarcoid-like reaction in lymph node of bile duct adenocarcinoma. No discernible histopathological differences were observed between CD205-positive- and CD205-negative granulomas. CD68 was consistently positive in granuloma cells. Double staining for CD205 and CD68 revealed that CD205^+^ cells also expressed CD68, confirming the expression of CD205 in granuloma cells (Fig. 1F–H). Ordway *et al*.^14^ and Welsh *et al*.^15^ reported that foamy macrophages in the periphery of granulomas expressed CD205. We did not observe the foamy appearance of granuloma cells in any samples tested.

### Distribution of T cells, B cells, and DCs

Granuloma cells were frequently (but not always) surrounded by a lymphocyte collar (Fig. 1C), which included T cells in all cases (Fig. 2C) and B cells that were usually sparser than T cells. B cells were present in greater numbers than T cells in lymphocyte collars in two cases (tuberculous lymphadenitis and BCG-treated urothelial carcinoma). T cells also infiltrated among the granuloma cells in all cases examined (Fig. 2C,G), and were termed intra-granuloma T cells. It was noteworthy that granulomas lacking a lymphocyte collar showed the same phenotype; i.e. granuloma cells expressed CD205 and harbored T cells (Fig. 2E–G). The immune granuloma cells uniformly expressed HLA-DR (Fig. 2B), and they usually lacked the expression of CD83, which was expressed by mature, conventional (myeloid-derived) DCs distributed in the lymphocyte collar (Fig. 2D). In five cases, CD83 was occasionally expressed by granuloma cells (Fig. S1C).

### Lack of CD205 expression in foreign body granulomas

Foreign body granulomas (14 cases) were analyzed as controls for the immune granulomas. Eleven cases of foreign body granulomas lacked CD205 expression irrespective of their size (Fig. 2H,I). It was notable that the CD205 expression was focally observed (5–20%) in the other three cases, only where lymphocytic infiltration was observed (Fig. S3A,B). This suggests that foreign body granuloma may show a phenotype similar to that of APC when inflammation is complicated.

### Intra-granuloma T cells

**Figure 3:**
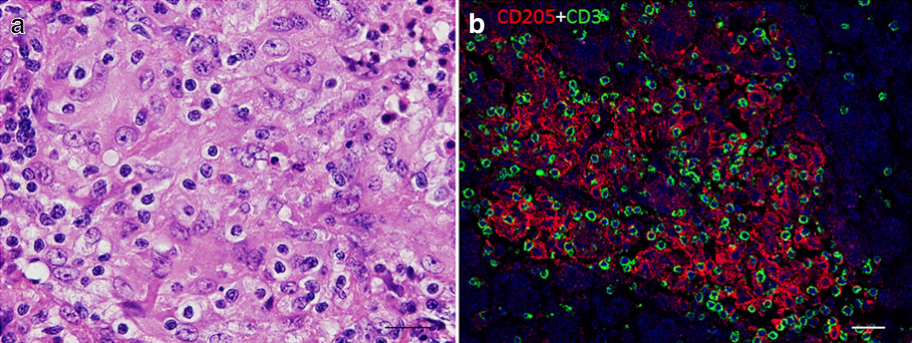
Intra-granuloma T cells. (**a**) High magnification (HE). Intra-granuloma lymphocytes are visible. (**b**) Double immunofluorescent staining clearly shows that CD205^+^ granuloma cells (red) harbor CD3^+^ T cells (green) in a case of hepatoma associated with multiple granulomas in cancer tissue. Scale bars: 20 μm in (**a**) and 30 μm in (**b**).

Immune granuloma cells consistently harbored intra-granuloma T cells. Higher magnification confirmed its presence by HE staining (Fig. 3a). To visualize the presence of intra-granuloma T cells, double staining of CD3 and CD205 was performed (Fig. 3b), and demonstrated that T cells (green) were distributed among CD205^+^ granuloma cells (red). CD4^+^ cells predominated over CD8^+^ T cells in or around granulomas (Fig. S2). We previously reported direct cell-to-cell contact between granuloma cells expressing B7-2 (CD86) and lymphocytes in Crohn's disease by immunoelectron microscopy.^7^ This previous finding is consistent with the intra-granuloma T cells described here.

### Ki-67 expression by intra-granuloma T cells

**Figure 4:**
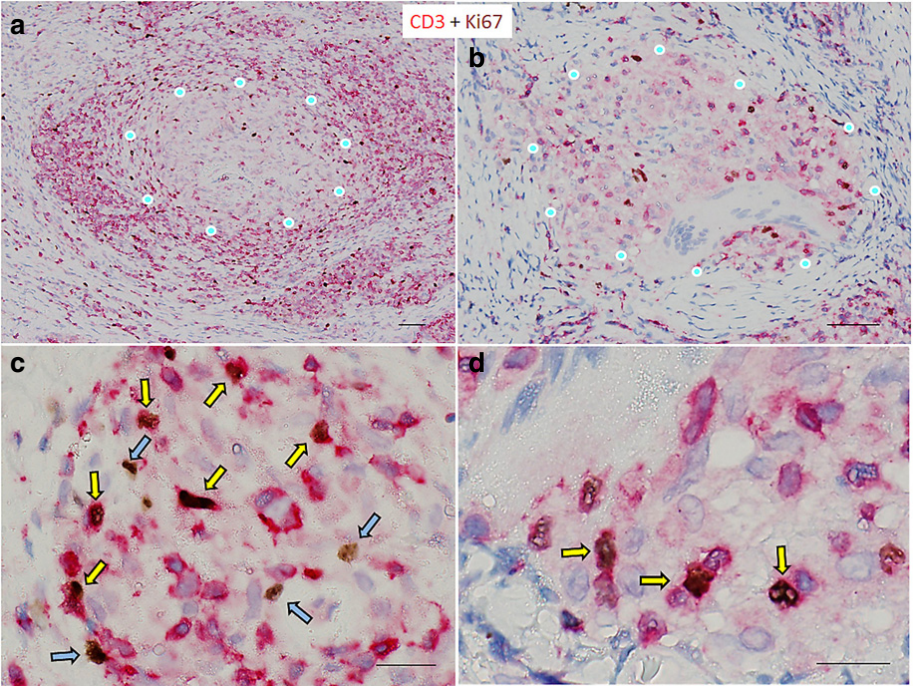
Proliferative activity in intra-granuloma T cells as revealed by double staining for CD3 and Ki-67. CD3 (red) and Ki-67 (brown) staining in a granuloma (**a**) with and (**b**) without lymphocyte collar. High magnification of a granuloma (**c**) with and (**d**) without lymphocyte collar. Note the similar occurrence of CD3^+^Ki-67^+^ cells (yellow arrows) located among granuloma cells. Blue arrows indicate Ki-67-single positive cells. Blue dots in **a** and **b** indicate the area of granuloma cells. Scale bars: 50 μm in (**a, b**) and 20 μm in (**c, d**).

The results thus far suggested that immune granuloma cells are phenotypically similar to (conventinal) DCs except for the infrequent expression of CD83. This suggests that granuloma cells may function as APCs. If this assumption is correct, lymphocytes within or near granulomas are expected to have a certain level of proliferative activity in active inflammatory lesions. Therefore, Ki-67 labeling index in intra-granuloma T cells was analyzed, because T cells in the lymphocyte collars around granulomas may be stimulated by DCs distributed in the same area. Double labeled immunohistochemistry for CD3 and Ki-67 was performed in 17 representative cases as described in the Materials and Methods (Fig. 4a,c). Double positive cells expressed brown color in the nucleus (Ki-67) surrounded by a red rim (CD3 reactivity) (yellow arrows in Fig. 4c,d). Ki-67-negative, CD3^+^ T cells have a hematoxylin-positive nucleus surrounded by a red reaction (blue arrows in Fig. 4c). The labeling index was calculated as the percentage of the total number of Ki-67^+^ CD3^+^ cells per total number of CD3^+^ cells. The median labeling index of Ki-67 in intra-granuloma T cells was 9.4% (25th percentile, 8.3% and 75th percentile 12.5%)(range from 5.5% to 41.4%). This clearly showed that intra-granuloma T cells were proliferating, not static or quiescent.

However, the intra-granuloma T cells may have been stimulated by DCs in the lymphocyte collar. Therefore, granulomas lacking the T cell-rich collar were carefully observed using specimens of double staining for CD3 and Ki-67. In 12 of 17 cases, granulomas not surrounded by a T cell collar were confirmed (Fig. 4b), which contained intra-granuloma T cells with Ki-67 reactivity (Fig. 4d). It is highly probable that only granuloma cells could stimulate intra-granuloma T cells to proliferate in granulomas not surrounded by T cell collar because of absence of nearby DCs that are expected to be distributed in lymphocyte collar.

### Intra-granuloma T cells express a higher labeling index of Ki-67 than T cells in the lymphocyte collar (peri-granuloma T cells) or T cells in lymphoid tissues

**Figure 5:**
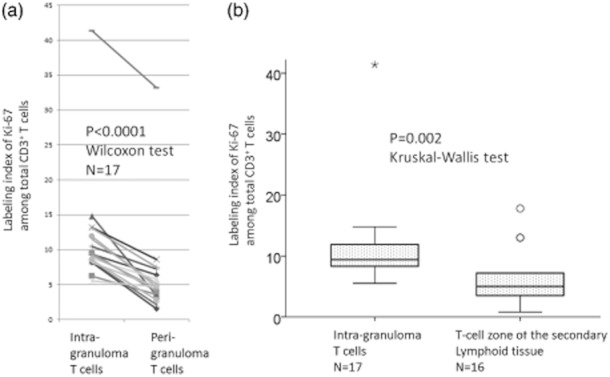
Comparative analysis of the labeling index of Ki-67 among CD3^+^ T cells. (**a**) Intra-granuloma T cells showed higher labeling index than T cells distributed in the lymphocyte collar (peri-granuloma T cells) (*P* < 0.0001). (**b**) Intra-granuloma T cells also show higher labeling index of Ki-67 than T cells in the paracortex of chronic tonsillitis and lymphadenitis (*P* = 0.002) (Box-whisker plot).

First, the Ki-67 labeling index was compared between intra-granuloma T cells and T cells in the lymphocyte collar (termed as ‘peri-granuloma T cells’) in the same case. As shown in Figure 5a, intra-granuloma T cells showed a higher labeling index of Ki-67 than peri-granuloma T cells (*P* < 0.0001). Next intra-granuloma T cells were compared with T cells in the paracortex (T cells zone) of the secondary lymphoid tissues (chronic tonsillitis and lymph nodes showing reactive hyperplasia). The same double staining for CD3 and Ki-67 was performed in these lymphoid tissues. As shown in Figure 5b, the labeling index of Ki-67 among T cells was higher in intra-granuloma T cells than in T cells in the T cell zone in secondary lymphoid tissues (*P* < 0.002). These data indicated that the labeling index of Ki-67 in intra-granuloma T cells was relatively high.

## Discussion

The present study shows clearly that the immune granuloma cells in granulomatous inflammation frequently express CD205 together with HLA-DR, irrespective of disease specificity. This is contrasted by the lack of CD205 expression in foreign body granulomas. Furthermore, the presence of intra-granuloma T cells was observed among immune granuloma cells, which express significant levels of Ki-67. This suggests that intra-granuloma T cells have a certain level of proliferative stimulation. The results here indicate a significant similarity between granuloma cells and APCs.

Granulomas are thought to be formed to contain offending agents that are difficult to eradicate, and are generally considered to be a protective response in terms of infectious disease. Tuberculosis is the most intensively analyzed disease among granulomatous inflammation, in which both the innate and adaptive immune responses are involved in the host defensive responses.^2^–^5^,^20^ However, a recent study demonstrated that *Mycobacterium tuberculosis* utilizes the granulomatous reaction to facilitate its spread.^21^,^22^ Their observations were based on the early innate immune response phase. Human clinical specimens from tuberculosis patients are usually obtained during the adaptive immune response phase, where lymphocytic infiltrates are frequently observed and lymphoid follicle-like structures are formed around granulomas.^23^ These adaptive immune responses are conceived to be protective.^2^,^22^,^23^ Therefore, in terms of human tuberculosis, it is reasonable to consider that granulomas may be functioning as host defense mechanisms.

Adaptive immune responses by T cells occur vigorously in granulomatous inflammation. DCs in granulomatous inflammation are important for the priming of T cells.^4^,^5^ CD83^+^ mature DCs are frequently located in granulomas in experimental studies^4^ and in human granulomas (lymphocyte collars) as revealed in the present study. In tuberculosis, foamy ‘macrophages’ in the lung express a DC phenotype including CD205.^15^ Together with DCs, the present study suggests for the first time that granuloma cells may function as APCs. High levels of Ki-67 expression in the intra-granuloma T cells suggest they receive a certain level of antigenic stimuli. We previously used this method to demonstrate immunological stimulus of T cells in histological sections.^18^,^19^ In the present study, comparative analysis between intra-granuloma T cells and T cells from the T cell zone (paracortex) of lymphoid tissue (chronic inflammatory changes) was performed. This revealed that the proliferative activity of intra-granuloma T cells was significantly higher compared with T cells from secondary lymphoid tissue in a chronically stimulated condition. The present study thus underlies the importance of cell kinetic analyses in granulomatous inflammation, since immune cell kinetics has begun to be investigated in the study of tuberculosis.^20^

These findings may also be important in diseases other than tuberculosis. Intra-bladder infusion of BCG is used to treat superficially-spreading urothelial carcinoma of the bladder. It is thought that non-specific activation of the innate and adaptive immune system could enhance anti-tumor immunity.^24^ Therefore, the present observation could help clarify the mechanisms of anti-tumor immunity. Crohn's disease is one of the two components of inflammatory bowel disease, in which non-caseating granuloma is a hallmark of disease. It is thought that a defect of cytokine secretion by macrophages (immunodeficiency of innate immunity) may cause ineffective bacterial clearance and subsequent abnormal T cell responses,^25^,^26^ which differentiate to Th1 rather than Th17 responses.^27^ The present results could be in accordance with these findings. Primary biliary cirrhosis is an autoimmune disease that targets intrahepatic small bile ducts, and is mediated in part by adaptive and innate immune cells.^28^ Granulomas are observed in the vicinity of the small bile ducts. The findings presented here may also help understand the pathophysiology of primary biliary cirrhosis. The mechanisms of granulomatous responses in tumors have not been elucidated. However, recent analyses showed that tumor-infiltrating lymphocytes, at least in some cancers, might function as an immunological defense against cancer cell proliferation.^29^,^30^

To conclude, the data here indicate a similarity between immune granuloma cells and APCs. Considering the active immune responses occurring in various granulomatous inflammation, the similarity permits us to speculate that immune granuloma cells themselves (particularly in the early phase) may function as APCs. The immune granuloma cells, thus, can cooperate with DCs distributed in the lymphocyte collar—a situation similar to the ‘T cell-DC cluster’ as postulated by Katou *et al*.^31^ Analytical methods will be required in future studies.

## SUPPORTING INFORMATION

**Figure S1** Immunohistochemical characterization of a granuloma in a case of tuberculous pleuritis in an exudative area. HE (**A**) and immunohistochemistry (brown color) for CD205 (**B**), CD83 (**C**) and CD3 (**D**). Differentiation of epitheloid cells is incomplete in this granuloma (early phase). The expression of CD83 is extensive here, particularly in giant cells. Note expression of CD205 and presence of intra-granuloma T cells. Blue dots indicate the area of granuloma cells. Scale bars: 50 μm.

**Figure S2** Immunohistochemical characterization of a granuloma in a case of tuberculous bursitis. HE (**A**) and immunohistochemistry (brown stain) for CD205 (**B**), CD4 (**C**) and CD8 (**D**). Note that CD4^+^ cells predominate over CD8^+^ cells among intra-granuloma T cells. CD205 is clearly expressed in most granuloma cells. Scale bars: 50 μm.

**Figure S3** Immunoshitochemistry for CD205 in a case of foreign body reaction (epidermal cyst) associated with chronic inflammation. HE (**A**) and CD205 immunostain (**B**). This granuloma is situated in the vicinity of epidermal cyst. Note that mononuclear foreign body granuloma cells occasionally express CD205 where lymphocytes are distributed. Scale bars: 100 μm.
