# Supplementary figures and images for: Granuloma cells in chronic inflammation express CD205 (DEC205) antigen and harbor proliferating T lymphocytes: Similarity to antigen-presenting cells
Source: Pathol Int. 2013 Feb 22;63(2):85–93. doi: 10.1111/pin.12036 (PMC3618377; doi:10.1111/pin.12036)

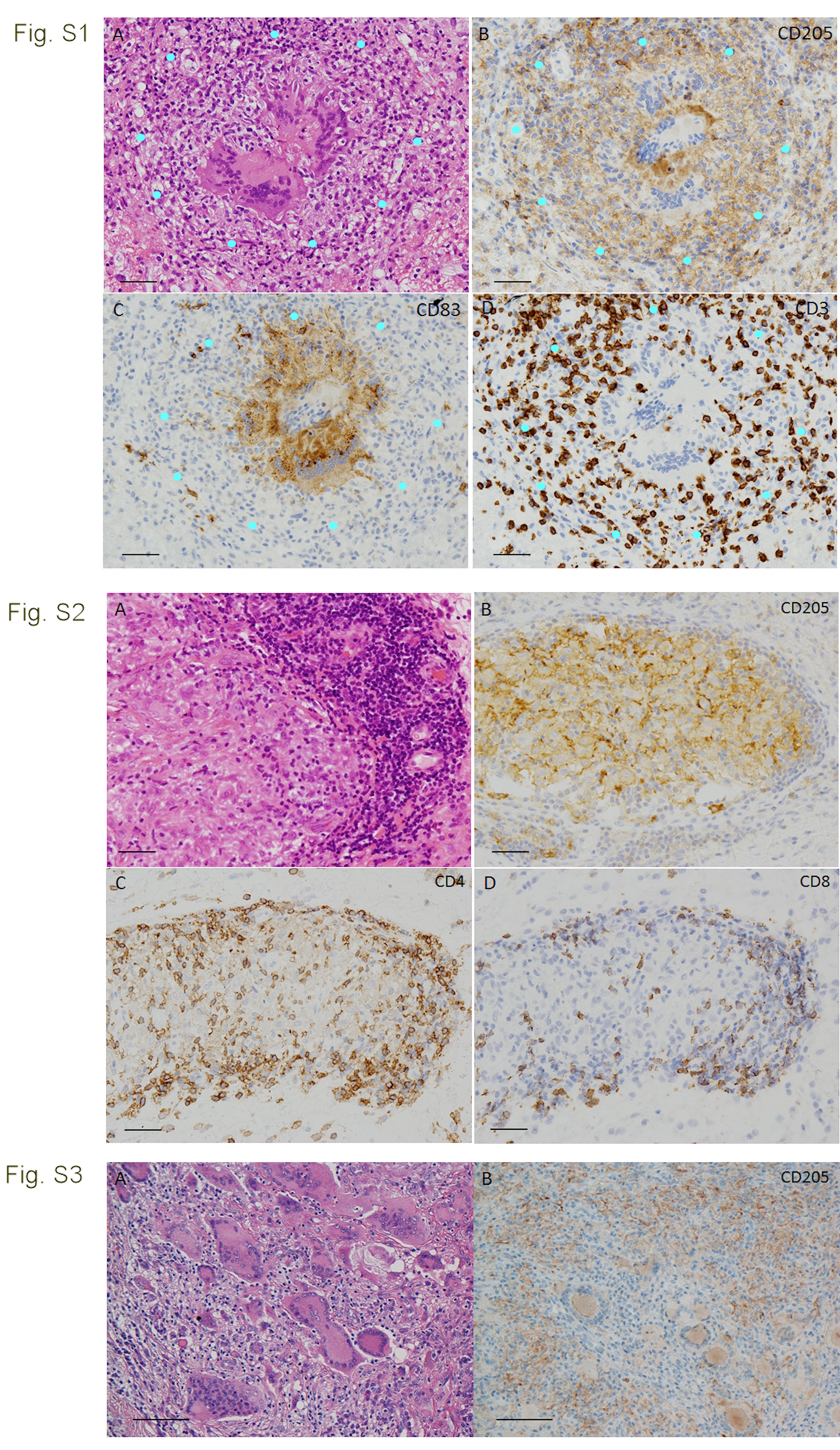

Supplement: Supplementary file 1 [file pin0063-0085-SD1.tiff]
